# Supplementary material for: Overweight and obesity among Vietnamese school-aged children: National prevalence estimates based on the World Health Organization and International Obesity Task Force definition
Source: PLoS One. 2020 Oct 12;15(10):e0240459. doi: 10.1371/journal.pone.0240459 (PMC7549813; doi:10.1371/journal.pone.0240459)
Supplement: S1 Fig — (PDF) [file pone.0240459.s001.pdf]

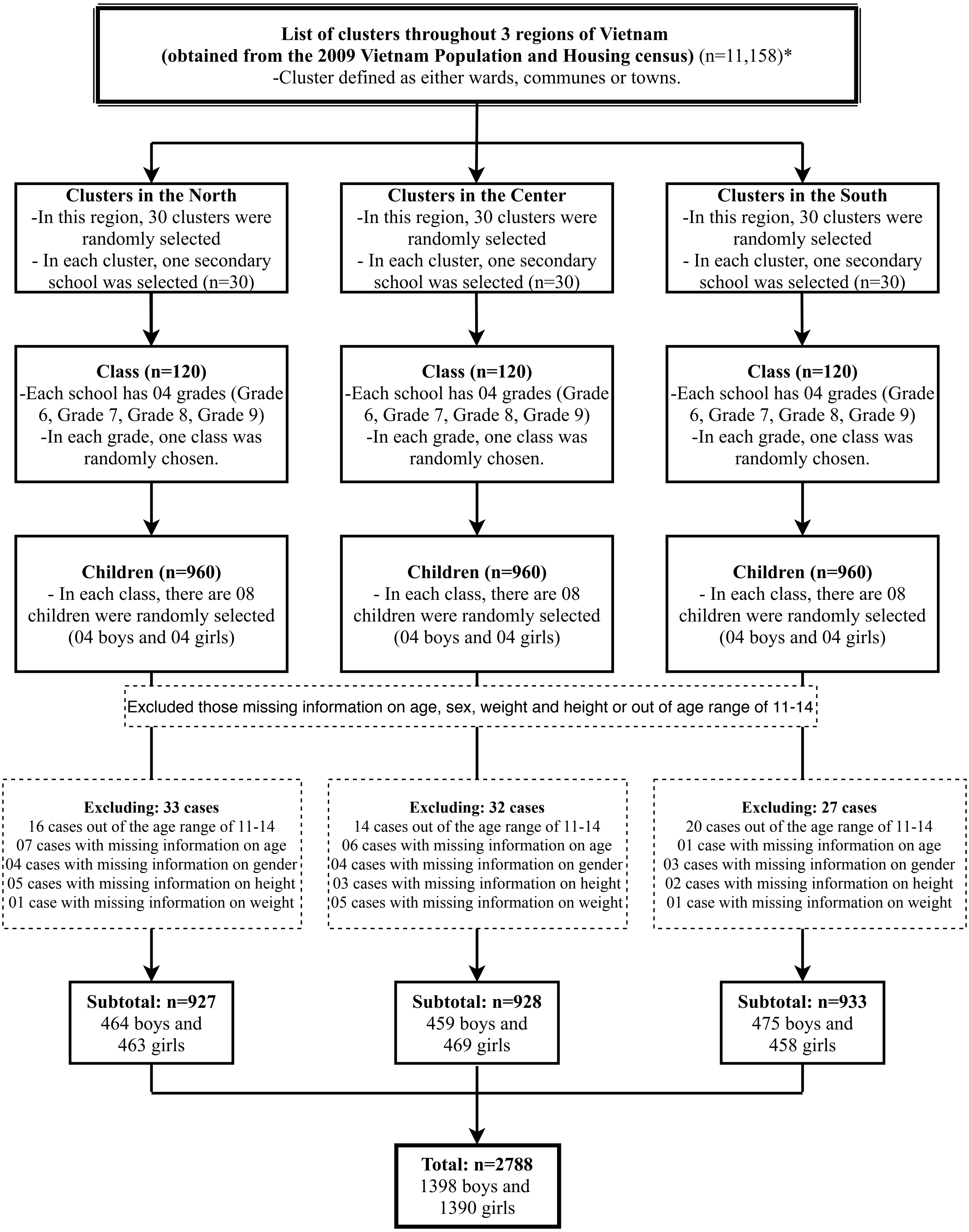

*General statistics office of Vietnam. The 2009 Vietnam Population and Housing census. 2009. (https://www.gso.gov.vn/default.aspx?tabid=512&idmid=5&ItemID=10438)
