# Supplementary material for: Overweight and obesity among Vietnamese school-aged children: National prevalence estimates based on the World Health Organization and International Obesity Task Force definition
Source: PLoS One. 2020 Oct 12;15(10):e0240459. doi: 10.1371/journal.pone.0240459 (PMC7549813; doi:10.1371/journal.pone.0240459)
Supplement: S1 Table — (PDF) [file pone.0240459.s002.pdf]

S1 Table. Overweight and obesity prevalence by age and sex based on four BMI metrics including WHO Z-score, WHO percentile, IOTF and CDC references

	Age	Boys			Girls			Total#		
		**WHO - Z-score**	**CDC**	**IOTF**	**WHO - Z-score**	**CDC**	**IOTF**	**WHO - Z-score**	**CDC**	**IOTF**
**Overweight**	11	20.7	30.1	23.2	15.6	16.5	15.1	18.2	23.4	19.2
**Obesity**		17.7	5.8	9.1	5.4	2.3	3.7	11.6	4.1	6.4
**Overweight**	12	22.7	30.7	25.3	14.8	16.2	14.5	18.7	23.3	19.8
**Obesity**		17.8	5.7	8.6	4.2	1.7	2.8	10.9	3.7	5.7
**Overweight**	13	19.4	19.7	16.4	15.9	16.8	15.1	17.7	18.2	15.8
**Obesity**		6.7	3.2	5.1	3.4	1.1	2	5.1	2.2	3.6
**Overweight**	14	14.2	17.4	14.2	15	19.6	12.8	14.6	18.5	13.5
**Obesity**		9.5	5.4	7.6	4	0.6	4.7	6.7	3	6.1

#Total was rounded up from the percentage of boys and girls

WHO Z-score: equivalent to 1SD-2SD for the overweight group and ≥2SD for obesity

CDC: defines overweight as 85th ≤BMI< 95th percentiles, and obesity as BMI ≥95th percentile

IOTF: equivalent to 25-30 kg/m^2 for the overweight group and ≥30 kg/m^2 for obesity at the age of 18
