# Supplementary material for: Overweight and obesity among Vietnamese school-aged children: National prevalence estimates based on the World Health Organization and International Obesity Task Force definition
Source: PLoS One. 2020 Oct 12;15(10):e0240459. doi: 10.1371/journal.pone.0240459 (PMC7549813; doi:10.1371/journal.pone.0240459)
Supplement: S2 Table — (PDF) [file pone.0240459.s003.pdf]

S2 Table. Summary of previous findings in Vietnamese children aged 11-16 years old

Year	Author	Study design	Location	Sampling method	Criteria	Overweight prevalence	Obesity prevalence
2007	Hong K Tang et al. [16]	A cross-sectional survey	Ho Chi Minh City	1504 Adolescents aged 11-16 years, mean age of 13.1	IOTF	-Boys: 5.8%<br>-Girls: 4.1%<br>-Total: 4.9%	-Boys: 0.9%<br>-Girls: 0.3%<br>-Total: 0.6%
2010	Tang KH et al. [13]	A cross-sectional survey	Ho Chi Minh City	2 678 students aged 11-16 from 31 junior high schools	IOTF	-Boys: 16.2%<br>-Girls: 7.2%<br>-Total: 11.7%	-Boys: 3.1%<br>-Girls: 1.0%<br>-Total: 2.0%
2010	Trang HHD Nguyen et al. [17]	A cross-sectional survey	Ho Chi Minh City	693 high-school students from urban districts, mean age of 13.9 (13.7-14.2 years)	IOTF	-Boy: 20.5% overweight/obese<br>-Girls: 11.4% overweight/obese<br>-Total: 15.5% overweight/obese	
2010	The United Nations in Vietnam [12]	A cross-sectional survey	Nation wide	Children and adolescents aged 6-19 years	WHO Z-score	-Boys: 13.0% (aged 11-13 years)<br>-Girls: 6.0% (aged 11-13 years)<br>-Total: 8.5% (children aged 6-19)	-Boys: Not found<br>-Girls: Not found<br>-Total: 0.7% (children aged 6-19)
2013	Huong Thi Le et al. [15]	A nested case-control study	Bac Giang	1528 secondary students (11-14 years old) selected from 16 secondary schools	WHO Z-score	-Boys: 9.6%<br>-Girls: 3.9%<br>-Total: 6.7%	-Boys: 2.8%<br>-Girls: 1.2%<br>-Total: 2.0%
2013	Phuong Van Ngoc Nguyen et al. [14]	A cross-sectional survey	Ho Chi Minh City	1,989 students aged 11–14 years in HCM	IOTF, WHO Z-score	-Boys: 22.0% (IOTF)<br>-Girls: 13.3% (IOTF)<br>-Total: 17.8% (IOTF), 19.6% (WHO Z-score)	-Boys: 5.4% (IOTF)<br>-Girls: 1.3% (IOTF)<br>-Total: 3.2% (IOTF), 7.9% (WHO Z-score)
2013	Hong TK et al. [18]	A prospective cohort study	Ho Chi Minh City	759 secondary high school students from 18 schools in urban districts.	IOTF	-Boy: 16.9% - 21.4% (over 5 years)<br>-Girl: 8.6% - 12.4% (over 5 years)	-Boy: 2.3% - 7.1% (over 5 years)<br>-Girl: 1.0% - 3.4% (over 5 years)

		(2004-2009)				-Total: 12.5% - 16.7% (over 5 years)	-Total: 1.7% - 5.1% (over 5 years)
2016	Nguyen PV et al. [19]	A cross-sectional survey	Ho Chi Minh City	2024 junior high school students aged 11-14 in all the 14 urban districts	IOTF	-Boy: 28.3% overweight/obese<br>-Girls: 14.5% overweight/obese<br>-Total: 21.1% overweight/obese	
2018	To QG et al. [20]	A cross-sectional survey	Ho Chi Minh City	619 students in public schools in urban areas, mean age was 10.4 years (9.7 to 12.6)	WHO Z-score	-Total: 52.7% were overweight/obese	
2018	This present study	A cross-sectional survey	Nation wide	2788 children aged 11-14 years	IOTF, WHO Z-score	-Boys: 19.4% (WHO Z-score), 19.9% (IOTF)<br>-Girls: 15.3% (WHO Z-score), 14.4% (IOTF)<br>-Total: 17.4% (WHO Z-score), 17.1% (IOTF)	-Boys: 12.9% (WHO Z-score), 7.6% (IOTF)<br>-Girls: 4.2% (WHO Z-score), 3.2% (IOTF)<br>-Total: 8.6% (WHO Z-score), 5,4% (IOTF)
