# Supplementary material for: Overweight and obesity among Vietnamese school-aged children: National prevalence estimates based on the World Health Organization and International Obesity Task Force definition
Source: PLoS One. 2020 Oct 12;15(10):e0240459. doi: 10.1371/journal.pone.0240459 (PMC7549813; doi:10.1371/journal.pone.0240459)
Supplement: S3 Table — (PDF) [file pone.0240459.s004.pdf]

S3 Table. Children' height by age and sex

Groups	Boys (cm)	Girls (cm)	Total (cm)
**11 years old**			
Mean (SD)	144.45 (8.38)	145.58 (7.54)	145.01 (7.99)
Median (IQR)	144.00 (139.00, 150.00)	146.00 (140.00, 151.00)	145.00 (140.00; 150.13)
**12 years old**			
Mean (SD)	151.87 (9.12)	149.93 (6.54)	150.88 (7.97)
Median (IQR)	152.00 (146.00, 158.00)	150.00 (146.00, 154.00)	151.00 (146.00; 156.00)
**13 years old**			
Mean (SD)	157.22 (8.43)	153.17 (5.87)	155.23 (7.56)
Median (IQR)	158.00 (151.00, 164.00)	153.00 (149.50, 157.00)	155.00 (150.00; 160.00)
**14 years old**			
Mean (SD)	161.82 (7.84)	154.27 (6.77)	158.02 (8.23)
Median (IQR)	162.00 (157.00, 167.00)	155.00 (150.00, 159.00)	158.00 (153.00; 163.00)
