# Supplementary material for: Overweight and obesity among Vietnamese school-aged children: National prevalence estimates based on the World Health Organization and International Obesity Task Force definition
Source: PLoS One. 2020 Oct 12;15(10):e0240459. doi: 10.1371/journal.pone.0240459 (PMC7549813; doi:10.1371/journal.pone.0240459)
Supplement: S4 Table — (PDF) [file pone.0240459.s005.pdf]

S4 Table. Prevalence of stunting and thinness by age and sex

Groups	Stunting		Thinness	
	**n (%)**	**95% CI**	**n (%)**	**95% CI**
**Boys**				
11 years old, n (%)	19 (5.2)	3.22-8.43	34 (9.4)	6.51-13.36
12 years old, n (%)	21 (6.0)	3.68-9.74	27 (7.8)	5.20-11.42
13 years old, n (%)	24 (6.5)	4.08-10.12	29 (7.8)	5.44-11.11
14 years old, n (%)	21 (6.6)	4.12-10.48	31 (9.8)	6.94-13.61
**Girls**				
11 years old, n (%)	27 (7.7)	5.15-11.28	34 (9.7)	6.79-13.56
12 years old, n (%)	30 (8.4)	5.43-12.65	21 (5.8)	3.67-9.21
13 years old, n (%)	22 (6.1)	4.00-9.34	22 (6.1)	4.09-9.13
14 years old, n (%)	31 (9.7)	6.15-14.84	18 (5.6)	3.60-8.63
**Total**				
11 years old, n (%)	46 (6.4)	4.62-8.92	68 (9.5)	7.15-12.58
12 years old, n (%)	51 (7.2)	5.00-10.31	48 (6.8)	4.76-9.59
13 years old, n (%)	46 (6.3)	4.60-8.60	51 (7.0)	5.40-9.01
14 years old, n (%)	52 (8.2)	5.63-11.67	49 (7.7)	5.82-10.07

Stunting (<-2 SD height-for-age WHO Z-score) and Thinness (<-2SD weight-for-height WHO Z-score)
