# Supplementary material for: Overweight and obesity among Vietnamese school-aged children: National prevalence estimates based on the World Health Organization and International Obesity Task Force definition
Source: PLoS One. 2020 Oct 12;15(10):e0240459. doi: 10.1371/journal.pone.0240459 (PMC7549813; doi:10.1371/journal.pone.0240459)
Supplement: S1 File — (PDF) [file pone.0240459.s006.pdf]

PHỤ LỤC 1. PHIẾU KHÁM

(do điều tra viên điền)

I. THÔNG TIN CHUNG

1. Thời gian khám : ....…… / …....… / 2018
2. Vùng/khu vực :... Mã số: [ ][ ]
3. Tỉnh/thành phố:.. Mã số : [ ][ ]
4. Quận/huyện : .... Mã số : [ ][ ]
5. Xã/phường: .... Mã số : [ ][ ]
6. Trường THCS: .... Mã số : [ ][ ]

Bạn có có đồng ý trả lời cho khảo sát này không? Đồng ý [ ] Từ chối [ ]

II. THÔNG TIN HỌC SINH

STT	CÂU HỎI	TRẢ LỜI	MÃ	CHUYỂN
7	Họ và tên	....	[ ] [ ]	
8	Ngày tháng năm sinh	....../…../20…		
9	Giới tính	Nam<br>Nữ	1<br>2	
10	Dân tộc	Kinh<br>Khác (ghi rõ:......................)	1<br>2	

III. THĂM KHÁM

STT	SỐ ĐO		GHI CHÚ
17	Chiều cao (cm)	[ ] [ ][ ],[ ]	
18	Cân nặng (kg)	[ ] [ ][ ],[ ]	
24	Tỷ lệ phần trăm mỡ cơ thể BFP (%)	[ ][ ],[ ]	

PHỤ LỤC 2. PHIẾU PHỎNG VẤN

(do điều tra viên điền)

I. THÔNG TIN CHUNG

1. Thời gian khám : ......… / …....… / 2018

2. Vùng/khu vực :.. Mã số:

3. Tỉnh/thành phố:... Mã số :

4. Quận/huyện : .... Mã số :

5. Xã/phường: .... Mã số :

6. Trường THCS: .... Mã số :

A. BỘ CÂU HỎI

1. Thông tin đối tượng được điều tra.

TT	NỘI DUNG	THÔNG TIN	MÃ	CHUYỂN
7	Họ và tên	....	[ ] [ ]	
8	Ngày tháng năm sinh	....../…../20…		
9	Giới tính	Nam<br>Nữ	1<br>2	
10	Dân tộc	Kinh<br>Khác (ghi rõ:......................)	1<br>2	

4. Thực hành của đối tượng được điều tra đối với phòng chống bệnh ĐTĐ trẻ em

4.2. Chế độ luyện tập và hoạt động thể lực (Đọc thông tin cho đối tượng được hỏi).

TT	CÂU HỎI	TRẢ LỜI	MÃ	CHUYỂN
11	Hàng ngày em đến trường bằng phương tiện gì?	Đi bộ<br>Đi xe đạp<br>Đi xe bus<br>Bố mẹ đưa đi<br>Phương tiện khác	1<br>2<br>3<br>4<br>5	
12	Ở nhà em thường giúp đỡ bố mẹ công việc gì?	Quét nhà<br>Rửa bát<br>Lau nhà<br>Giặt quần áo<br>Không phải làm gì<br>Khác (ghi rõ):...........	1<br>2<br>3<br>4<br>5<br>6	
13	Em thường chơi môn thể thao gì?	Cầu lông<br>Nhảy dây	1<br>2	

		Bóng bàn<br>Bóng đá<br>Đá cầu<br>Bơi<br>Khác (ghi rõ):...........	3<br>4<br>5<br>6<br>7	
14	Em thường chơi bao lâu?	...................ngày/tuần<br>...................phút/ngày		
15	Em có chơi điện tử không?	Có<br>Không	1<br>2	
16	Em thường chơi bao ngày/tuần ?	.................ngày<br>/tuần		
17	Một ngày em chơi điện tử mấy giờ?	<=1 giờ<br>1 giờ<br>2 giờ<br>3 giờ<br>>=4 giờ	1<br>2<br>3<br>4<br>5	
18	Em có xem tivi không?	Có<br>Không	1<br>2	
19	Em thường xem bao ngày/tuần ?	.................ngày<br>/tuần		
20	Một ngày em xem tivi mấy giờ?	<=1 giờ<br>1 giờ<br>2 giờ<br>3 giờ<br>>=4 giờ	1<br>2<br>3<br>4<br>5	
21	Em có sử dụng mạng xã hội (facebook, zalo...)?	Có<br>Không	1<br>2	
22	Em thường sử dụng bao lâu?	...................ngày/tuần<br>...................phút/ngày		
23	Em có đi học thêm không?	Có<br>Không	1<br>2	
24	Nếu có mức độ học thêm như thế nào?	1 buổi/tuần.<br>2 buổi/tuần<br>3 buổi/tuần.<br>4 buổi/tuần<br>≥ 5 buổi buổi/tuần	1<br>2<br>3<br>4<br>5	

Điều tra viên

(Chữ ký, họ tên)

PHỤ LỤC 3. PHIẾU TỰ ĐIỀN

PHIẾU TỰ ĐIỀN ĐIỀU TRA DỊCH TỄ HỌC BỆNH ĐÁI THÁO ĐƯỜNG VÀ CÁC YẾU TỐ NGUY CƠ Ở TRẺ EM LỨA TUỔI 11-14 TUỔI TOÀN QUỐC NĂM 2018

(dành cho phụ huynh)

I. THÔNG TIN CHUNG

1. Thời gian khám : ....…… / …....… / 2018

2. Vùng/khu vực :.. Mã số:........

3. Tỉnh/thành phố:... Mã số :.........

4. Quận/huyện : .... Mã số :..........

5. Xã/phường: .... Mã số :.........

6. Trường THCS: .... Mã số :........

Bạn có có đồng ý trả lời cho khảo sát này không? Đồng ý ☐ Từ chối ☐

Chữ kí của người được điều tra

II. PHẦN PHỎNG VẤN

TT	CÂU HỎI	TRẢ LỜI	MÃ	CHUYỂN
7	Họ và tên phụ huynh	....	[ ] [ ]	
8	Họ và tên học sinh	....		
9	Trình độ học vấn phụ huynh	Tiểu học<br>THCS<br>THPT<br>THCN/CĐ/ĐH<br>Trên ĐH	1<br>2<br>3<br>4<br>5	
10	Nghề nghiệp phụ huynh	Cán bộ nhà nước<br>Công nhân<br>Nông dân<br>Buôn bán<br>Nội trợ<br>Nghề khác	1<br>2<br>3<br>4<br>5<br>6	
11	Cân nặng Anh/chị	Bố: ....,........ kg<br>Mẹ: ....,...... kg		
12	Chiều cao của anh/chị	Bố: ......,....... m<br>Mẹ: ....,...... m		

13	Anh chị có mắc bệnh Đái tháo đường không?	Có (Anh hoặc chị mắc)<br>Cả 2 đều mắc<br>Không	1<br>2<br>3	
14	Anh/chị có bị tăng huyết áp không?	Có (Anh hoặc chị mắc)<br>Cả 2 đều mắc<br>Không	1<br>2<br>3	
15	Anh/chị đã từng bị rối loạn mỡ máu không	Có (Anh hoặc chị mắc)<br>Cả 2 đều mắc<br>Không	1<br>2<br>3	

SECTION 1: PHYSICAL MEASUREMENT

(filled by data collectors)

IV. GENERAL INFORMATION

1. Date of interview : ......…. / …....… / 2018
2. Region :... Code: [ ][ ]
3. Province:.. Code: [ ][ ]
4. District : .... Code: [ ][ ]
5. Cluster: .... Code: [ ][ ]
6. Name of school: .... Code: [ ][ ]

Do you agree to answer this survey? Agree [ ] Disagree [ ]

V. STUDENT'S INFORMATION

No	QUESTION	ANSWER	CODE	SKIP
7	Full name	....	[ ] [ ]	
8	Date of birth	....../…../20…		
9	Gender	Boys<br>Girls	1<br>2	
10	Ethnicity	Kinh<br>Other (specify:......................)	1<br>2	

VI. MEASURE

No	MEASURE		NOTE
11	Height (cm)	[ ] [ ][ ],[ ]	
12	Weight (kg)	[ ] [ ][ ],[ ]	
13	Body fats percentage (%)	[ ][ ],[ ]	

SECTION 2. INTERVIEW
(filled by data collectors)

GENERAL INFORMATION

1. Date of interview : ......… / ….....… / 2018

2. Region :... Code:		
3. Province:... Code:		
4. District : .... Code:		
5. Cluster: .... Code:		
6. Name of school: .... Code:		

Do you agree to answer this survey? Agree [ ] Disagree [ ]

Personal information

No	QUESTION	ANSWER	CODE	SKIP
7	Full name	....	[ ] [ ]	
8	Date of birth	....../…../20…		
9	Gender	Boys<br>Girls	1<br>2	
10	Ethnicity	Kinh<br>Other (specify:......................)	1<br>2	

Behavior information

No	QUESTION	ANSWER	CODE	SKIP
11	What type of transportation that you use to go to school daily?	Walking<br>By bicycle<br>By bus<br>Parent assisstance<br>Other	1<br>2<br>3<br>4<br>5	
12	What type of chore works do you do at home?	Sweeping<br>Washing dishes<br>Cleaning the floor<br>Washing clothes<br>Nothing	1<br>2<br>3<br>4<br>5	

		Others (Specify:...............)	6	
13	What type of sports that you ussual play?	Badminton<br>Skipping<br>Table tennis<br>Soccer/Football<br>Shuttlecock kicking<br>Swiming<br>Khác (ghi rõ):............	1<br>2<br>3<br>4<br>5<br>6<br>7	
14	How long do you ussual play?	....................days/week<br>....................minutes/day		
15	Do you play video games?	Yes<br>No	1<br>2	
16	How long do you ussual play?	....................days/week		
17	How many hours that you usual play video games per day?	<1 hour<br>1 hour<br>2 hours<br>3 hours<br>>=4 hours	1<br>2<br>3<br>4<br>5	
18	Do you watching television?	Yes<br>No	1<br>2	
19	How long do you ussual watch TV?	....................days/week		
20	How many hours that you usual watch TV per day?	<1 hour<br>1 hour<br>2 hours<br>3 hours<br>>=4 hours	1<br>2<br>3<br>4<br>5	
21	Do you use social media (Facebook, Zalo,...)?	Yes<br>No	1<br>2	
22	How long do you ussual use?	....................days/week<br>....................minutes/day		
23	Do you take an extra class?	Yes<br>No	1<br>2	
24	If yes, how many extra classes do you have every week?	1 section/week<br>2 sections/week<br>3 sections/week<br>4 sections/week<br>≥ 5 sections/week	1<br>2<br>3<br>4<br>5	

Data collector

(Sign)

SECTION 3. SELF-REPORT QUESTIONNAIRE

(for student's parent)

----

1. Date of report : ..…..…. / ….….… / 2018

2. Region :..	Code:		
3. Province:..	Code:		
4. District :	Code:		
5. Cluster:	Code:		
6. Name of school:	Code:		

Do you agree to answer this survey? Agree ☐ Disagree ☐

Signature

No	QUESTION	ANSWER	CODE
7	What's your full name?	....	
8	What's your children'name?	....	
9	What is the highest education level that you completed?	Primary school<br>Secondary school<br>High school<br>Vocational/College/Univeristy<br>Postgraduate	1<br>2<br>3<br>4<br>5
10	What is your main occupation?	Government employee<br>Industry worker<br>Farmer<br>Self-employ<br>Home makers<br>Other (specify:.................................)	1<br>2<br>3<br>4<br>5<br>6
11	What are your partner's and your weight?	Farther: ....,........ kg<br>Mother: ....,...... kg	
12	What are your partner's and your height?	Farther: ......,....... m<br>Mother: ....,...... m	
13	Do you or your partner suffer from hypertension?	Yes, only one<br>Yes, both<br>No	1<br>2<br>3

14	Do you or your partner suffer from diabetes?	Yes, only one<br>Yes, both<br>No	1<br>2<br>3
15	Do you or your partner suffer from hyperlipidemia?	Yes, only one<br>Yes, both<br>No	1<br>2<br>3
