# Supplementary material for: Overweight and obesity among Vietnamese school-aged children: National prevalence estimates based on the World Health Organization and International Obesity Task Force definition
Source: PLoS One. 2020 Oct 12;15(10):e0240459. doi: 10.1371/journal.pone.0240459 (PMC7549813; doi:10.1371/journal.pone.0240459)
Supplement: Attachment — Submitted filename: A rebuttal letter.docx [file pone.0240459.s007.docx]

Dear Sir/Madam,

We would like to express our sincere thanks for the editor for your comments on our manuscript. We hope that a revised version of the manuscript will be considered for publication.

We have modified the paper in response to the insightful comments of the editor. Furthermore, we have rewritten sections of the manuscript and hope that this complies with the referee’s remarks.

We have responded to the comments point by point. Our answers to the editor’s questions were highlighted in yellow. All the line number mentioned were based on the revised manuscript.

****
PONE-D-20-04809
Overweight and obesity among Vietnamese school-aged children: National prevalence estimates based on the World Health Organization and International Obesity Task Force definition
PLOS ONE

Dear Dr. Bui,

Thank you for submitting your manuscript to PLOS ONE. After careful consideration, we have decided that your manuscript does not meet our criteria for publication and must therefore be rejected.

Specifically:
This is an important paper given the paucity of information available from Vietnam. The sampling strategy is very good.

Unfortunately, the cut-offs used for WHO 2007 growth reference used by you have a mistake which makes the analysis wrong. For the under-five, we use the WHO Anthro Software and here the overweight is defined as BAZ > +2SD and obesity is defined as BAZ > +3SD. But for children and adolescents 5-19 years of age we use WHO AnthroPlus software, and for this age group, BAZ >+1SD is considered a threshold for overweight and BAZ >+2SD is considered the threshold for obesity. This is very well explained in the paper by de Onis (your reference 10 in the paper). Also you can go through the manual for Anthro Plus software (Available from: <https://www.who.int/growthref/tools/who_anthroplus_manual.pdf>). You may also read the references 29-38 used in this manuscript to better understand this. Reference 31 clearly mentions this criteria.

Thank you for your precious and very detailed comment. In this revised manuscript, we made several changes according to your comments as follows:

- We redefined the diagnostic criteria according to WHO: overweight 1-2 SD, obese >=2SD (<https://www.who.int/growthref/growthref_who_bull.pdf>) (described in section **Criteria for childhood overweight and obesity**, line 143-144)
- We utilized the WHO 2007 Stata package of WHO to calculate BMI for age (variable “_zbfa”) (<https://www.who.int/growthref/tools/readme_stata.pdf?ua=1>). Age is calculated by subtracting date of birth from date of anthropometric measurement (described in section **Survey instruments and physical measurement**, line 127-128).
- We also installed the WHO AnthroPlus software (<https://www.who.int/growthref/tools/who_anthroplus_manual.pdf>) and randomly double checked 20 cases to make sure the results obtained from the WHO 2007 Stata package matched with the results from the WHO AnthroPlus software. All estimates were similar.
- After correcting the calculation, we obtained the new prevalence of overweight and obesity by using the WHO Z-score criteria (described in section **Criteria for childhood overweight and obesity**, line 142-144). This prevalence of overweight and obesity in Vietnamese children were 17.4% and 8.6%, respectively by WHO Z-score criteria.
- We also re-ran the model and re-estimated the association between the socio-demographic characteristic and the childhood overweight and obesity. Our findings still showed that parental BMI was associated with a higher prevalence of being overweight and obese in children, regardless of sex. Older age and belong to ethnic minority group were associated with an increased prevalence of childhood overweight and obese among boys also However, watching TV from 3 hours per day or more was no longer a significantly associated factor (described in **Figure 2 and Figure 3,** line 255-269).

Thank you very much for your consideration. We are looking forward to your comments and suggestions.

Yours sincerely,

Linh Phuong Bui, MD, MPH
